# Effectiveness of a novel long-lasting pyriproxyfen larvicide (SumiLarv®2MR) against *Aedes* mosquitoes in schools in Yangon, Myanmar

**DOI:** 10.1186/s13071-017-2603-9

**Published:** 2018-01-06

**Authors:** Sai Zaw Min Oo, Sein Thaung, Yan Naung Maung Maung, Khin Myo Aye, Zar Zar Aung, Hlaing Myat Thu, Kyaw Zin Thant, Noboru Minakawa

**Affiliations:** 1 Department of Vector Ecology and Environment, Institute of Tropical Medicine, Nagasaki University, 1-12-4 Sakamoto, Nagasaki, 852-8523 Japan; 2 Graduate School of Biomedical Sciences, Nagasaki University, 1-12-4 Sakamoto, Nagasaki, 852-8523 Japan; 3 Department of Medical Research, Ministry of Health and Sports, 11191, Ziwaka Road, Dagon Township, Yangon, Myanmar

**Keywords:** Pyriproxyfen, SumiLarv®2MR, Larvicide, Dengue, *Aedes*, Schools, Myanmar

## Abstract

**Background:**

Mosquito-borne diseases are prevalent in Myanmar, with the number of dengue cases showing a significant increase in recent years. Dengue vectors have developed resistance to insecticides and currently used larvicides show only short-term effectiveness. As a result, an alternative larvicide is urgently needed. The present study evaluated the larvicidal effectiveness of long-lasting pyriproxyfen resin discs (SumiLarv®2MR) against dengue virus vectors in schools in Hlaing Thar Yar Township, Yangon.

**Results:**

The proportion of *Aedes* mosquito-infested containers was significantly reduced in the schools applied with the larvicide (OR: 0.24, 95% CI: 0.12–0.48) while there was little reduction noted in the control schools (OR: 0.97, 95% CI: 0.55–1.72). The density of infested containers was also significantly reduced in the intervention schools (Beta: -1.50, 95% CI: -1.98– -1.04), but there was no significant reduction in density in the control schools (Beta: -0.19, 95% CI: -0.53–0.14). The proportion of adult emergence was less than 20% in the treated water collected from the intervention schools for six months, while the proportion was over 90% in the untreated water. In addition, eight-month-old SumiLarv®2MR resin discs were still 100% effective when tested in the laboratory. More than 50% of the discs disappeared from treated containers within two months of intervention.

**Conclusions:**

SumiLarv®2MR was effective in reducing *Aedes*-infested containers at least six months after its application in schools. This new pyriproxyfen formulation has great potential for improving the current dengue vector control program in Myanmar.

## Background

Myanmar, as with many tropical countries, is subjected to many vector-borne diseases such as malaria, lymphatic filariasis, Japanese Encephalitis, dengue, and chikungunya [1] and, most recently, Zika virus [2]. In recent years, there has been a significant increase in the number of Myanmar-based dengue cases. In 2015, the Ministry of Health and Sports (MOHS) reported more than 40,000 dengue cases, which exceeded the previous record for the highest number of cases (24,285) reported in 2009 (MOHS, unpublished data). Regardless of the continuous efforts to control vectors, on a yearly basis, dengue fever is of the most concern to both local health care workers and residents.

A dengue vaccine is a potential global game changer [3, 4], particularly when used as recommended by the WHO in combination with well-executed and sustained vector control, evidence-based best practices for clinical care for all patients with dengue illness, and strong dengue monitoring [5]. The primary control activity in Myanmar targets the larval stages of dengue virus vectors; temephos (1% *w*/w sand granules) is the larvicide currently being used for vector control [6, 7]. Temephos has been used extensively in Thailand, Cambodia and Columbia for many years, resulting in larvicide resistance by *Aedes* mosquitoes [8–11]. Although temephos resistance was not detected in the laboratory tests among *Aedes* mosquitoes collected from Mandalay and Yangon, Myanmar in 2013 and 2014 (Yi Yi Mya and Yan Naung MM, personal communication), an alternate dengue vector control measure was noted as necessary to alleviate reliance on temephos and to minimize further selection of resistant phenotypes.

In 2015, Sumitomo Chemical Company Limited (Tokyo, Japan) introduced a novel larvicide formulation (SumiLarv®2MR). SumiLarv®2MR is a long-lasting matrix release formulation (5.5 cm diameter containing 2% *w*/w pyriproxyfen) that is, designed for container breeding mosquitoes [12, 13]. Pyriproxyfen has a unique mode of action that affects the metamorphosis, reproduction and embryogenesis of insects, as well as inhibiting adult emergence from pupae. As a result, death typically occurs at the pupal stage [13–15]. An advantage of pyriproxyfen is that it requires a lower concentration than other larvicides such as temephos and *Bacillus thuringiensis israelensis* (*Bti*) [6]. SumiLarv®2MR is formulated as a small resin disc using slow release technology that can maintain effectiveness for over six months [12, 16, 17]. Although pyriproxyfen has not been widely used in Myanmar, a trial in Yangon found that a larvicide based on pyriproxyfen (SumiLarv®0.5G) successfully reduced the number of *Aedes* mosquitoes [18]. SumiLarv®2MR, however, includes the advantage of its time release capability and resulting effectiveness duration over SumiLarv®0.5G [15–17].

The main objective of this study was to evaluate the effectiveness of SumiLarv®2MR against *Aedes* mosquitoes breeding in containers within schools. Public places such as schools and parks are widely known to be locations where significant dengue virus transmission can occur [19, 20]. In particular, the chance for children to be infected may be high at schools, because *Aedes aegypti* is a day-time biter [21, 22], and school-aged children spend a large part of their day at schools. Results of past studies, however, have been inconsistent [23–26].

## Methods

### Study area

The study area was established in Hlaing Thar Yar Township (16°51′0″N, 96°4′0″E), one of the most populated townships (the population was approximately 700,000) in Yangon, Myanmar. A total of 373 dengue cases were reported from the township in 2014. In 2015, the incidence of dengue increased to 409 cases, and 257 (63%) of these were school children between 5 to 16 years of age. The numbers were highest among the townships in Yangon (MOHS 2015, unpublished data).

Twelve out of 50 public schools were selected for the present study based on a programmed enrollment age between 5 and 16 years. In choosing the programmed age criterion, the selected 12 schools were also the largest in the study township. Approximately 40,000 students were enrolled in the selected schools during the study period, representing more than half of the total students in the township. The mean distance between each pair of selected schools was 3403 m (SE = 220.3, ranged from 690 to 7710 m), and the schools were far enough away from each other to avoid the spillover effects of the average *Ae.aegypti* flight range of < 200 m [27, 28].

### Advocacy meeting.

An advocacy meeting was held in May 2015 to explain the research in detail to the teachers, stake-holders and township authorities. After the meeting, the information sheets describing the larvicide were posted on the walls of all school buildings.

### Characteristics of aquatic habitats

Baseline surveys were conducted to characterize potential breeding containers in the schools in June, August, September, October and November 2015 (pre-intervention period). A potential breeding container was defined for the study as a container filled with water and accessible by adult mosquitoes for ovipositing. Each container type was categorized into the barrel, bucket, cement tank, cement drum, ceramic jar, discarded container, spiritual bowl or toilet bowl. Containers sizes were categorized into small (< 10 l), medium (10–200 l) or large (> 200 l). Locations of containers were also classified as indoor or outdoor. Subsequently, a field worker inspected each container for the occurrence (presence or absence) of immature *Aedes* mosquitoes using a flashlight for 2 to 5 min (the searching time depended on type and size of the container, and water turbidity). Mosquitoes were left in-situ to avoid disturbing container habitats.

### Effects on occurrence of *Aedes* mosquitoes in containers

Twelve schools were further divided into two groups in which six schools were randomly selected for intervention, and six schools remained as a control. At the end of November 2015, SumiLarv*®*2MR resin discs were placed in all potential breeding containers in the intervention schools. Based on dosage recommendations, one disc was used for 40 l of water. Additional discs were placed in containers with a volume larger than 40 l or, conversely, a disc was cut smaller depending on container volume. When multiple discs were placed in one container, they were tied together with a wire. Students and school staff members were asked not to remove the discs. In December 2015 (one month after the application), each container was inspected for the occurrence of *Aedes* mosquitoes and the presence/absence of discs. Containers in the control schools were also examined for *Aedes* mosquitoes. This survey was repeated in January, March and May 2016. In each survey, the occurrence of the discs was also recorded for each container.

### Residual effects of treated water

Water (400 ml) was sampled from each of the randomly selected ten containers in the intervention schools in November 2015 before placing the larvicide. Each water sample was stored in a 500 ml plastic container (height = 11 cm, diameter = 8 cm). Each plastic container was then sealed and labeled. Ten water samples were also collected from the control schools. Any visible life forms were removed from the water sample using a pipet. On the same day that water was sampled, 20 laboratory-reared fourth-instar larvae of *Ae.aegypti* were placed in each container in the laboratory. The larvae were F2 and F3 generations of a laboratory colony established from larvae originally collected in the study area. The containers were placed in a control chamber with 27–30 °C and 60% humidity after adding powdered mouse-food pellets (Laboratory Animal Services Division, Department of Medical Research, Yangon, Myanmar) with a food concentration of 10 mg/l. Each container was checked daily until all individuals emerged or died. The adult emergence total was recorded and the proportion of adult emergence (the number of adults emerged divided by 20) was estimated. The experiment was repeated four times during the post-intervention period December 2015, and January, March and May 2016. Water samples were collected from the randomly selected containers that had discs in the intervention schools at each survey point.

### Effectiveness of eight-month-old resin discs

Eight months after the application, any resin discs that remained in the containers were collected. From the remaining discs, five were randomly selected and placed individually in 45 l plastic containers filled with 40 l of tap water. Prior to the experiment, the containers with tap water were screened with a net and placed in a dark room for three days to condition the water. Twenty-four instar larvae (F2 and F3 generations) of laboratory-reared *Ae.aegypti* were then placed in each container, and powdered mouse-food pellets (a concentration of 10 mg/l) were added. Each container was screened with a net and placed in a control chamber (27–30 °C with 60% humidity). The containers were checked daily until all individuals emerged or died; the adult emergence total was recorded. For control, the same procedure was repeated for containers without a disc. The experiment was repeated once.

### Statistical analysis

The generalized linear mixed-effects model (GLMM) was used to test whether the proportion of containers infested with immature *Aedes* mosquitoes was significantly different among three container sizes during the pre-intervention period. Similarly, the occurrences of *Aedes* mosquitoes were compared between indoors and outdoors. Survey point (month) and school were considered random factors. A caterpillar plot (*ggplot 2* package, R version 3.3.3) was tested to whether each of the random factors was necessary.

GLMM was also used for impact assessment, comparing the proportions of infested containers tallied during the post-intervention period (December 2015 to May 2016) with those of the pre-intervention period (June to November 2015). The data were analyzed separately for the intervention schools and the control schools. The proportions were also compared between the intervention schools and the control schools. Similarly, the impact of SumiLarv*®*2MR resin discs was assessed for the density of infested containers (the number of infested containers per 100 m^2^). The geographical coordinates of each school were recorded using a handheld global positioning system (GPSMAP®62 s, Garmin Ltd., Kansas, US). The Google Maps Area Calculator Tool (Draft logic version 6.9) was used to estimate the area (m^2^) of each school. The area estimation included school buildings, staff residential areas in the school campus, detached toilets, detached canteens, corridor areas around buildings, outdoor water storage tanks and rain-water collected containers areas. However, bare playgrounds were excluded. In these analyses, month and school were also considered as random factors.

To assess the residual effects of SumiLarv*®*2MR discs in the field, the proportion of infested containers in the intervention schools at each post-intervention survey point was compared with the baseline proportion of November 2015 using GLMM. This analysis only considered data from the containers that had water. The analysis excluded data from the containers that lost water or disappeared even once during the period. Containers that lost discs were included in the analysis. The proportion of infested containers in the control schools at each post-intervention survey was also compared with the November 2015 baseline proportion. In the same way, the proportion of adult emergence in the treated water collected at each survey point was compared with the baseline proportion in November 2015 using GLMM. The emergence data from the water samples in the control schools were also analyzed with GLMM. The surveyed school was considered a random factor in the analyses.

## Results

### Characteristics of *Aedes-*infested sites

A total of 1376 potential breeding containers were recorded in 12 schools during the pre-intervention period, and *Aedes* mosquitoes were found in 273 containers (19.8%). Among the eight habitat types, cement drums were found to be the most infested containers, followed by discarded containers and toilet bowls (Table 1). While 88% of discarded containers were infested, less than 40% of other types of containers were infested.

**Table 1:** Characteristics and numbers (%) of infested containers found in 12 selected schools during the pre-intervention period

Container		Mean volume (range) (l)	No. of potential breeding containers	No. of infested containers (%)	Percentage based on total infested containers
Type	Barrel	167.5 (50–400)	111	25 (22.5)	9.2
	Bucket	29.8 (10–100)	128	13 (11.7)	4.8
	Cement drum	149.7 (5–800)	519	79 (15.2)	28.9
	Cement tank	987.4 (20–2000)	121	14 (11.6)	5.1
	Ceramic jar	93.8 (50–150)	98	21 (21.4)	7.7
	Discarded container	8.7 (1–150)	64	53 (87.5)	19.4
	Spiritual bowl	1.0 (1)	55	16 (34.5)	5.9
	Toilet bowl	14.9 (5–50)	280	44 (15.7)	16.1
Size	Large (>200 l)	1039.8 (250–2000)	127	13 (10.2)	4.8
	Medium (10–200 l)	97.9 (10–200)	1029	178 (17.3)	65.2
	Small (<10 l)	3.5 (1–5)	220	82 (37.3)	30.0
Place	Indoor	55.1 (1–2000)	394	63 (16.0)	23.1
	Outdoor	215.8 (1–2000)	982	210 (21.4)	76.9
Total		169.8 (1–2000)	1376	273 (19.8)	100

Barrels and ceramic jars were locally manufactured, and their sizes were standardized to 50, 100, 150, and 200 l. The sizes of cement drums were also standardized by local manufacturers, except four drums that were smaller than 10 l. Cement tanks were customized by owner and the sizes varied from 20 to 2000 l. All spiritual bowls and most discarded containers were smaller than 10 l. Nearly three-quarters of toilet bowls were in the middle size range. Categorizing the containers by size, 65% of the infested containers were of medium size, but more than a third of small containers were infested. The binomial GLMM revealed that the proportion of infested small containers was significantly higher than the others (OR: 4.41, 95% CI: 2.82–6.96 when compared with medium containers; OR: 12.01, 98% CI: 5.74–26.94 when compared with large containers). Nearly three-quarters of the infested containers were found outdoors, but the difference in proportion of infested containers was not statistically significant between indoor and outdoor (OR: 0.96, 95% CI: 0.66–1.39). School and month were included as random factors in the analysis.

### Fate of discs

Immediately after the baseline survey in November 2015, SumiLarv®2MR discs were placed in 111 containers that had water in the intervention schools. In December (one month after the intervention), 19 containers had dried up and were excluded from the survey. All 92 remaining containers still had water and discs. In January, 53 (57.6%) of the containers lost all discs. The number of containers that lost discs rose to 71 (77.2%) by March and 76 (82.6%) by May. All small containers, such as spiritual bowls and toilet bowls, lost their discs after six months. Discs also disappeared from all indoor containers by May (Table 2).

**Table 2:** Numbers (%) of containers with lost discs in each post-intervention survey

Container		*n*^a^ (baseline)	Mean volume (range) (l)	December	January	March	May^b^
Type	Barrel	7	135.7 (100–200)	0 (0)	0 (0)	3 (42.8)	4 (57.1)
	Bucket	5	60.0 (5–100)	0 (0)	2 (40.0)	4 (80.0)	4 (80.0)
	Cement drum	43	130.2 (50–300)	0 (0)	25 (58.1)	31 (72.1)	34 (79.1)
	Cement tank	6	650.0 (100–1000)	0 (0)	1 (16.6)	3 (50.0)	3 (50.0)
	Ceramic jar	1	50.0 (50)	0 (0)	0 (0)	1 (100.0)	1 (100.0)
	Discarded	2	5.5 (1–10)	0 (0)	1 (50.0)	2 (100.0)	2 (100.0)
	Spiritual bowl	10	1.0 (1)	0 (0)	7 (70.0)	9 (90.0)	10 (100.0)
	Toilet bowl	18	20.0 (20)	0 (0)	17 (94.4)	18 (100.0)	18 (100.0)
Size	Large (>200 l)	7	621.4 (250–1000)	0 (0)	1 (14.3)	3 (42.8)	3 (42.9)
	Medium (10–200 l)	74	92.2 (10–200)	0 (0)	44 (59.5)	58 (78.4)	62 (83.8)
	Small (<10 l)	11	1.0 (1)	0 (0)	8 (72.7)	10 (90.1)	11 (100.0)
Place	Indoor	30	22.3 (1–100)	0 (0)	27 (90.0)	29 (96.7)	30 (100.0)
	Outdoor	62	169.5 (1–1000)	0 (0)	26 (41.9)	42 (67.7)	46 (74.2)
Total		92	121.5 (1–1000)	0 (0)	53 (57.6)	71 (77.2)	76 (82.6)

^a^Number of containers that had water continuously from November (after removing 19 containers that lost water or disappeared by December)

^b^Although six new containers with discs appeared in May, they were excluded because they were not original containers that received discs in November

### Effects on proportion of infested containers

The cumulative total number of potential breeding containers was 685 in the intervention schools and 691 in the control schools during the pre-intervention period (from June to November 2015). During the same period, the cumulative total number of *Aedes*-infested containers was 187 in the intervention schools and 86 in the control schools. The proportion of infested containers was 27.3% in the intervention schools, and 12.5% in the control schools during that period. The binomial GLMM revealed that the intervention schools had a significantly higher proportion of infested containers than the control schools (OR: 2.99, 95% CI: 1.14–7.82).

During the post-intervention period (from December 2015 to May 2016), the cumulative total number of potential breeding containers was 374 in the intervention schools and 481 in the control schools. The cumulative total number of *Aedes*-infested containers was 35 in the intervention schools and 57 in the control schools. The proportions of infested containers in the intervention schools and control schools were 9.4% and 11.9%, respectively. The difference was not significant (OR: 0.70, 95% CI: 0.20–2.48).

When the proportions of infested containers in the intervention schools were compared between the pre-intervention period and post-intervention period, the proportion was significantly smaller for the post-intervention period (OR: 0.24, 95% CI: 0.12–0.48). For the control schools, the proportion of infested containers was not significantly different between pre-intervention and post-intervention periods (OR: 0.97, 95% CI: 0.55–1.72). Only the school variable was considered as a random factor when the proportion was compared between the intervention schools and the control schools during the post-intervention period because the caterpillar plot did not show clear effects of different survey points. The other models included both school and month variables as random factors.

### Effects on density of infested containers

**Fig. 1:**
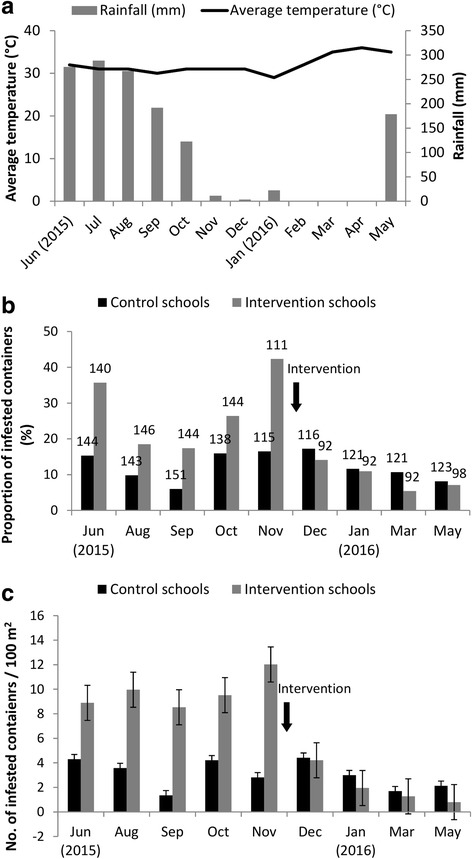
**a** Average temperature (°C) and rainfall (mm), **b** proportions of infested containers (the number on each bar shows the total number of containers inspected) and **c** numbers of infested containers per 100 m^2^ during the study period

The density of infested containers was 6.5 ± 1.0 (mean ± SE) containers/100 m^2^ in the schools during the pre-intervention period. In the intervention schools and control schools, the density was 9.8 ± 1.7 containers/100 m^2^ and 3.3 ± 0.5 containers/100 m^2^, respectively. The Poisson GLMM revealed that the difference was statistically significant (Beta: 1.09, 95% CI: 0.28–1.94). After the intervention, the density of infested containers became 2.1 ± 0.4 containers/100 m^2^ in the intervention schools, and 2.8 ± 0.6 containers/100 m^2^ in the control schools. Since the distribution of data was over dispersed, a GLMM with negative binomial distribution was used to test the difference in post-intervention density between the intervention schools and control schools. The difference was not statistically significant (Beta: -0.28, 95% CI: -1.37–0.84). When the post-intervention density in the intervention schools was compared with the pre-intervention density, it was significantly less (Beta: -1.50, 95% CI: -1.98–-1.04). For the control schools, the post-intervention density was not significantly lower than that of the pre-intervention period (Beta: -0.19, 95% CI: -0.53–0.14) (Fig. 1).

### Residual effects

As water was present in 92 containers in the intervention schools at all survey points during the period from November 2015 to May 2016, the data from these containers were used to compare the proportion of infested containers at each of the four post-intervention survey points with the baseline proportion in November 2015. In November, the proportion of infested containers was 35.9%; the number was reduced to 5.4% after six months (Table 3). The binomial GLMM revealed that the difference in the proportion of infested containers between November and each of the post-intervention survey point was statistically significant. During the same period, water was present in 115 containers in the control schools. The proportion of infested containers in the control schools was 16.5% in November, which decreased to 7.0% after six months. The proportion in May was significantly lower than that in November, while the proportion in November was not significantly different from proportions in the earlier months.

**Table 3:** Changes in the number (%) of infested containers in intervention schools and control schools

Survey point	Control schools			Intervention schools		
	*n*	Infested (%)	Odds ratio (95% CI)	*n*	Infested (%)	Odds ratio (95% CI)
November^a^	115	19 (16.5)	1	92	33 (35.9)	1
December	115	19 (16.5)	1 (0.47–2.22)	92	13 (14.1)	0.28 (0.11–0.65)^*^
January	115	10 (8.7)	0.45 (0.18–1.03)	92	10 (10.9)	0.21 (0.08–0.52)^*^
March	115	12 (10.4)	0.56 (0.24–1.24)	92	5 (5.4)	0.10 (0.03–0.27)^*^
May	115	8 (7.0)	0.34 (0.13–0.83)*	92	5 (5.4)	0.10 (0.03–0.27)^*^

^*^P < 0.05; significant difference when compared with the baseline proportion of November

^a^Before the intervention (baseline)

The residual effects of treated water were assessed in the laboratory. For the water collected from the containers in the intervention schools, the baseline proportion of adult emergence in November 2015 was 97.5%. This proportion was reduced to less than 10% for the treated waters collected at the first three post-intervention survey points (Table 4), a value significantly lower than the baseline proportion.

**Table 4:** Adult emergence numbers (%) from water samples collected from both control schools and intervention schools

Survey point	Control schools			Intervention schools		
	*n*	Adults (%)	Odds ratio (95% CI)	*n*	Adults (%)	Odds ratio (95% CI)
November^a^	200	194 (97.0)	1	200	195 (97.5)	1
December	200	196 (98.0)	1.01(0.83–1.23)	200	3 (1.5)	0.02 (0.00–0.04)^*^
January	200	189 (94.5)	0.97 (0.80–1.19)	200	4 (2.0)	0.02 (0.01–0.05)^*^
March	200	198 (99.0)	1.02 (0.84–1.24)	200	18 (9.0)	0.09 (0.05–0.15)^*^
May	200	195 (97.5)	1.01 (0.82–1.23)	200	38 (19.0)	0.19 (0.14–0.27)^*^

^*^P < 0.05; significant difference when compared with the baseline proportion of November

^a^Before the intervention (baseline)

After six months, the proportion increased to nearly 20%, but it was still significantly lower than the baseline proportion. In the water from the control schools, the proportion of adult emergence was 97% in November and remained almost same after that. Although the proportions were not significantly different between the intervention schools and control schools in November, the proportions for the intervention schools became significantly lower after that.

All mosquitoes (*n* = 200) died in the water treated with eight-month-old discs within 72 h while a total of 189 larvae (94.5% adult emergence, *n* = 200) became adults in control water during the same period.

## Discussion

The proportion of infested containers in the intervention schools was reduced by one-third after the application of SumiLarv®2MR resin discs, while the proportion remained almost unchanged in the control schools. Similarly, the density of infested containers was reduced by 25% in the intervention schools, while the reduction was slight in the control schools. The seasonal effects may explain the slight reduction in the control schools; the dry season started concurrently with the application (Fig. 1). Despite the random selection of intervention and control schools, the intervention schools had a higher proportion and density of infested containers during the pre-intervention period. This was likely due to the small sample size of schools. Nevertheless, these results clearly showed the effects of the larvicide. The results are comparable to the past field studies that tested the effectiveness of larvicides based on pyriproxyfen [18, 29, 30].

As pyriproxyfen does not kill immature mosquitoes upon initial exposure [14–16], the effects were not visible immediately, possibly lowering confidence in the larvicide [13]. However, the present study showed that the effects become visible within one month of application, indicating that the adult population was reduced enough to affect larval population within one month. A field trial of pyriproxyfen in Colombia also observed a clear change in the number of infested containers one month after the application [29].

The most important features of SumiLarv®2MR resin disc are its time release and longer lasting effects. According to the manufacturer, the effectiveness may persist over six months because of the resin controlled-release technology [12, 16]. Application of the larvicide is needed only twice a year, and the operational time and cost become less than temephos or *Bti*, both of which have shorter residual efficacy [6]. The present study confirmed the residual effects of discs under the field and laboratory settings. Although the effectiveness was slightly reduced after six months in the adult emergence experiment, the odds ratio with the treated water was still 0.19 when compared with the untreated water as a baseline. These results are comparable to the results from the studies in Cambodia that tested the effectiveness of a 5% controlled-release formulation [31]. In addition, eight-month old discs from the field had 100% inhibition of emergence, suggesting that the discs continued to release pyriproxyfen and work as well as a new disc. The SumiLarv®2MR resin disc also had more than 80% adult emergence inhibition up to 36 weeks in semi-filed and laboratory conditions in Laos [17]. Moreover, the formulation used in the study in Cambodia inhibited over 80% of emergence for 34 weeks at least [31]. These controlled-release devices have potential for acting for an even longer period than eight months. As the current vector control activity in Myanmar uses temephos which is effective for 8 to 12 weeks [6, 7], a vector control with SumiLarv®2MR can significantly reduce operational time and manpower.

The controlled-release resin disc has other some clear advantages over the other larvicides, including its stable concentration, as well as its easily recognizable size and colour. Because of the controlled-release technology, SumiLarv®2MR can maintain stable pyriproxyfen concentration even after water overflows from the container with rainfall. As the disc is visible because of its size and colour, field workers and residents can easily confirm whether the water has been treated and the disc is still present. In the study area, water in spiritual bowls and toilet bowls are small and clear enough to see the discs. As water in the other containers except discarded ones is mainly used for daily life, the water is usually clear enough to recognize discs. When water is turbid, field workers should be able to confirm disc presence by searching using hands or a stick, a process not possible for granule type larvicide use. Moreover, field workers can easily estimate the amount of pyriproxyfen remaining in the container by counting the number of discs in the follow-up surveys. Using this method, overdosing the larvicide can be avoided, thus reducing operational costs, as well as mosquito larvicide resistance. Cost savings can also be realized through the removal of discs by residents during container cleaning, then placing the disc back in the container. In contrast, sand granules that are used as mediums for pyriproxyfen and temephos are likely to be lost through the washing process [32].

Of concern in the present study was the high number of missing discs. Although discs were present in all containers one month after application, over 50% of them disappeared within two months. As the school authorities and local health workers were told that the survey team would revisit the school one month after the application, there was a tendency to remove or hide containers with missing discs before the container inspection. To avoid this bias, the dates of follow-up surveys were not announced in advance. In the present study, smaller containers such as spiritual bowls were more likely to lose discs, which may have been due to frequent replacement of water in these containers. Moreover, school teachers reported that some children removed discs from the containers to play and did not return them. This issue was noted as a potential child-based curiosity that overrode instructions given by teachers at the outset of the research. Posters and verbal instructions were not enough to avoid losing discs, especially in schools. These results suggest the importance of deeper community involvement and understanding by participants [18, 33]. Given the importance of effective communications with the study communities, COMBI (Communication-for-Behavioral-Impact) will be useful in planning the social communication, participation, and understanding in vector control activities [13, 34].

### Limitation

Although the proportion and density of infested containers were significantly reduced in the intervention schools, the small sample size of schools compromised generalizability. As the current study targeted all school-aged children (5 to 16 years old), the number of schools was limited in the area. The number of data collectors required for an increased number of schools was also limited. Although school children reportedly removed the discs from the containers, further study is needed to confirm the causes of missing discs, including both observing and interviewing children.

Plastic containers were used in the laboratory experiments that evaluated the residual effects. As plastic can absorb the active ingredients of pyriproxyfen and reduce the bioavailability [35], the results from the experiments might underestimate the effectiveness of the SumiLarv®2MR resin disc.

## Conclusions

SumiLarv®2MR resin discs reduced the *Aedes* mosquito population in the intervention schools. The effectiveness lasted for at least six months, possibly longer. This new pyriproxyfen formulation has a great potential for improving the current vector control program because implementing vector control activities in the townships of Yangon has been a big challenge for health authorities, especially for long-term sustainability.

## Abbreviations

CI: confidence interval
OR: odds ratio
SD: standard deviation
